# Therapeutic hypothermia in infants <36 weeks gestation: proceed with caution

**DOI:** 10.1038/s41390-026-04912-w

**Published:** 2026-03-11

**Authors:** Elizabeth Sewell, Atul Malhotra, Alistair J. Gunn

**Affiliations:** 1 Emory University School of Medicine & Children’s Healthcare of Atlanta, Atlanta, GA USA; 2 Department of Paediatrics, Monash University, Melbourne, VIC Australia; 3 Department of Physiology, University of Auckland, Auckland, New Zealand

Therapeutic hypothermia (TH) reduces the rate of death or disability in term and near-term infants with hypoxic-ischemic encephalopathy (HIE).^1^ Although studies in preterm animals suggested neuroprotection with TH,^2,3^ the initial human trials included limited numbers of infants at 35 weeks gestation and none at less than 35 weeks.^4,5^ Over time, more preterm infants have been treated with TH,^6^ despite sparse evidence of safety and efficacy of TH in this population. A large international survey found that 60% of sites use 35 weeks as the minimum gestational age for TH.^7^ Recently, a landmark trial by Faix et al enrolled 168 infants between 33 0/7 to 35 6/7 weeks gestation, randomized to TH versus normothermia, and found no benefit of TH on death or moderate or severe disability and a possible increase in mortality with TH.^8^ Faix and colleagues included 48 infants at 35 weeks gestation (of whom 28 were treated with TH). Although this is the largest randomized TH trial for preterm infants to date, it was not powered for subgroup analysis at different gestations,^8^ and there is still controversy, particularly around the impact on infants of 35 weeks gestation.^9^

The cohort study by Martinez et al. in this issue of *Pediatric Research* provides additional safety and efficacy data on TH in infants less than 36 weeks gestation.^10^ The aim of this study was to compare clinical, neuromonitoring, neuroimaging, and neurodevelopmental outcomes among infants with moderate or severe HIE born at 34 0/7–35 6/7 weeks gestation (preterm, *n* = 63) to 36 0/7–37 6/7 weeks gestation (near term, *n* = 59), all of whom received TH. This single center retrospective study included outborn infants born 34 0/7–37 6/7 weeks gestation with moderate or severe HIE with evidence of intrapartum compromise and moderate or severe encephalopathy on modified Sarnat examination. After adjusted for severity of encephalopathy, preterm infants 34–35 weeks gestation had greater odds of death (adjusted odds ratio (aOR) 3.49, 1.06–11.50; *p* = 0.04) and for the composite outcome of death or moderate or severe encephalopathy (aOR 2.94, 1.02–8.46; *p* = 0.046) compared to near term infants. Additionally, compared to near term infants, preterm infants were more likely to have hemodynamic instability (67% vs 32%, p < 0.001), hypoglycemia (54% vs 36%, *p* = 0.04), and had more severe white matter injury scores on MRI (7 vs 4, *p* = 0.003).

The main limitations of the present study are that it was retrospective, nonrandomized, and without a control group receiving normothermia.^8^ Since all infants were treated with TH, this study cannot distinguish between harm due to TH treatment from worse outcomes due to greater level of prematurity. Of note, only one previous cohort study included preterm infants with both TH and normothermia, and it was limited by its small sample size.^11^ Previous cohort studies comparing TH in preterm infants to term infants were limited by even smaller sample sizes and heterogenous near term comparison groups.^11–13^ Importantly, Martinez et al report neurodevelopmental outcomes after discharge, albeit 35% of infants were lost to follow-up. Importantly, only two previous cohort trials were able to include neurodevelopmental outcomes but were limited by small sample size or greater loss to follow up.^11,14^

There are several limitations of the present study that affect all with all studies of preterm hypoxia-ischemia. Prematurity itself can impact exam findings on modified Sarnat exam, particularly altered tone, posture, and reflexes. Due to these findings, the randomized trial by Faix et al accommodated some modified Sarnat components to account for prematurity and required included preterm infants to have an abnormal level of consciousness.^8^ This requirement of abnormal level of consciousness was included in the study by Roca-Llabres et al., but not by Martinez et al. and previous cohort studies.^11–14^ Further, unlike the trial by Faix et al., Martinez et al. and other cohorts did not require examiner training or certification,^8,11–14^ and used the Weeke classification, which has not been validated for infants <36 weeks gestation.^15^

Lastly, Martinez et al did not report temperature monitoring. Preterm infants are at greater risk for hypothermia than term infants due to higher surface area to body weight ratios and skin characteristics, and admission hypothermia has been associated with adverse outcomes.^16,17^ Excessive hypothermia has been associated with adverse outcomes in infants with HIE.^18^ It is noteworthy that 40% of preterm infants in the preterm trial for TH had excessive hypothermia, with more in the TH group compared to control.^8^ When these infants were excluded, there were no clinically significant differences between TH infants and controls.^8^ It is possible that newer models of servo-controlled cooling blankets with increased temperature sampling and adjustments may improve thermal stability during TH, but remains unclear if that will meaningfully alter outcome.

The rates of adverse outcomes reported by Martinez et al. are similar to those reported in the TH arm of the randomized trial by Faix et al., which found higher rates in infants treated with TH compared to normothermia.^8^ The use of composite outcomes in this and other studies enhances statistical power and avoids competing outcomes but complicated by unequal clinical significance of component outcomes.^19^ Previous studies suggest that parents prioritize survival over disability, but this has not been studied specifically after HIE.^20^ In this study and others of perinatal HIE,^21,22^ most deaths followed redirection of care, which may increase the risk of prognostic bias and obscure true treatment effects.

Current variability in specialty society guidelines reflects this uncertainty in the benefits of TH across GAs, with minimum ages for offering TH varying between 35, 36, and 37 weeks gestation.^23–29^ Taken together, the present study and all available evidence suggest that routine TH in infants <36 weeks gestation lacks demonstrated benefit and may confer harm. Until higher-quality data are available, routine use of TH in this population should be approached with caution, and where possible, contribute to research efforts. Importantly, the consistently high mortality observed across studies highlights a critical knowledge gap: whether adverse outcomes reflect physiologic intolerance to TH or downstream effects from intensive care. Addressing this gap will require focused investigation into mechanisms of brain injury and death in preterm infants with HIE, with the goal of determining precise definitions of phenotyping of encephalopathy and brain injury in preterm infants to guide safe, modifiable, evidence-based neuroprotective strategies.
